# Traffic Speed Prediction: An Attention-Based Method

**DOI:** 10.3390/s19183836

**Published:** 2019-09-05

**Authors:** Duanyang Liu, Longfeng Tang, Guojiang Shen, Xiao Han

**Affiliations:** College of Computer Science and Technology, Zhejiang University of Technology, Hangzhou 310023, China (D.L.) (L.T.) (X.H.)

**Keywords:** intelligent transportation system, traffic speed prediction, attention mechanism, temporal clustering analysis

## Abstract

Short-term traffic speed prediction has become one of the most important parts of intelligent transportation systems (ITSs). In recent years, deep learning methods have demonstrated their superiority both in accuracy and efficiency. However, most of them only consider the temporal information, overlooking the spatial or some environmental factors, especially the different correlations between the target road and the surrounding roads. This paper proposes a traffic speed prediction approach based on temporal clustering and hierarchical attention (TCHA) to address the above issues. We apply temporal clustering to the target road to distinguish the traffic environment. Traffic data in each cluster have a similar distribution, which can help improve the prediction accuracy. A hierarchical attention-based mechanism is then used to extract the features at each time step. The encoder measures the importance of spatial features, and the decoder measures the temporal ones. The proposed method is evaluated over the data of a certain area in Hangzhou, and experiments have shown that this method can outperform the state of the art for traffic speed prediction.

## 1. Introduction

Traffic speed prediction, especially short-term prediction (less than 20 min), has become increasingly important in intelligent transportation systems (ITSs) [1]. Many modern traffic facilities and applications rely heavily on the accuracy of prediction. For example, the navigation system can provide an optimal route for travelers based on real-time prediction, and can calculate the cost of travel time, which is helpful for making plans. The traffic speed can reflect the traffic state of the road network; based on the current value of traffic speed and its future short-term change trend, managers can partition the traffic network [2], optimize the signal timing, and guide the traffic traveling, so as to make full use of road resources and alleviate traffic congestion.

Because of the substantial amount of potential, traffic prediction has become a hot topic in the field of traffic over the past few decades. Considering that the current traffic states are relevant to the upstream and downstream roads, and are also similar to the same horizon of previous weekdays and weekends, various data-driven algorithms have been proposed to increase the prediction reliability and accuracy. In general, approaches can be categorized into three parts: parametric methods, non-parametric methods, and deep learning methods [3,4,5].

Parametric approaches rely on a fixed parameter set, assuming that the collected data satisfy a similar distribution. A most widely used approach is the auto-regressive integrated moving average model (ARIMA), which is a time-series prediction model and assumes that the data are stationary, that is to say, the mean value and variance remain unchanged. The ARIMA model was first used in traffic prediction in [6], and in the past few decades, a number of its extensions have been proposed [7,8]. Although such method is easy to implement, it can only be used in linear system. The traffic system is far more complex, if using such method, the prediction accuracy is not reliable enough.

Different from parametric methods, non-parametric methods are believed to more flexibly fit the nonlinear characteristics and are better able to process noisy data [9]. A Kalman filter is a main model to predict future states, which can be applied to any dynamic system whose state variables are uncertain. Kalman-filter-based approaches have been applied in traffic prediction in [10,11,12]. Other statistical techniques such as the hidden Markov model [13] and Bayesian interference [14] have also been under research in recent years. Recently, along with the arrival of big data, machine-learning-based methods have drawn academic attention. Davis and Nihan first applied the *k*-nearest-neighbors (*k*-NN) method to short-term traffic prediction, considering the stochastic traffic features. The results were comparable but were unable to reach the benchmarks [15]. One deficiency of *k*-NN is that the selection of *k* relies heavily on priori or experiential knowledge. Besides, as the historical data are growing every day, the search space becomes even more enormous, resulting in substantial costs when searching for an optimal solution. To resolve this problem, Simon et al. proposed an improved sequential search strategy with *k*-NN [16]. The support vector machine (SVM) and support vector regression (SVR) are considered as two other efficient algorithms for short-term traffic speed prediction. SVR maps multi-dimensional traffic data into a feature space and performs a linear regression within that space [17]. Several studies [18,19,20] have proved that SVR can outperform time-series methods. However, these studies with shallow architectures have been proved to have limitations in such a high-dimensional and complex traffic state [21].

With the enrichment of data and rapid development of data processing, neural network-based methods have become one of the most researched algorithms due to its capability of dealing with non-linear and multi-dimensional data [22,23,24,25]. Ma et al. utilized the long short-term memory (LSTM) structure to perform traffic speed prediction and obtained a higher accuracy compared to statistical or other classical methods [22]. Du et al. took multi-parameters as inputs, and presented a hybrid neural network that combined convolutional neural networks (CNNs) and LSTM to predict traffic [23]. However, the algorithms mentioned above do not consider spatial relations between roads or segments; that is to say, a certain road’s traffic speed is relevant to, or will be influenced by, its upstream and downstream roads’ speed. Zeng et al. incorporated the previous (temporal) inputs and exogenous (spatial) inputs to train a recurrent neural network (RNN) [26]. Li et al. used deep belief networks (DBNs) to predict short-term traffic flow using temporal-spatial traffic data, and a multi-objective particle swarm algorithm was used to optimize some of the parameters in certain networks [27]. Gu et al. captured the lane-level spatio-temporal characteristics traffic speed using the fusion deep learning (FDL) model [28]. Ma et al. converted the network traffic to images, and constructed a time–space matrix using time and space dimension data, which were collected from GPS devices, and a CNN-based network was finally introduced for prediction [22]. Yu et al. captured spatial-temporal features through graph convolution [29].

Furthermore, as traffic speed prediction can also be regarded as a sequence prediction task, research shows that the longer the length of sequences is, the lower the accuracy will be [30]. Consequently, an attention mechanism is processed to determine which part of the sequence is more important for improving prediction precision. As an attention mechanism has been demonstrated to have a better performance in many fields [31,32,33], a hybrid attention-based model with CNN to mine spatial features and a gated recurrent neural network (GRU) to mine temporal features was proposed [34]. GRU is a variant of RNN, which can model the long-term dependencies and vanishing error problem. The attention mechanism is aimed to measure the importance of the past inputs correlated to the future states. However, the space dimension still contains some temporal information, and the CNN model somewhat neglects the inner temporal relations. Liao et al. [35] applied an attention mechanism to predict traffic speed with map query data; however, it can only predict speeds around the busy roads, and was limited to the query data. Since there are various applications to queries, the integration may be a challenge.

To deal with the problems above, we introduce a novel traffic speed prediction approach based on temporal clustering and hierarchical attention (TCHA). Firstly, according to the time series information and the topological structure between the target road and the surrounding road, the historical data are divided into two parts: spatial data and temporal data. Secondly, to distinguish the traffic environment, temporal clustering analysis is applied to the target road [36], which separates the historical data into several clusters. Traffic data in each cluster have similar distribution, which can help improve the prediction accuracy. Thirdly, a hierarchical attention-based mechanism is used to measure the importance of each feature at each time step, the spatial attention in the encoder measures the importance of spatial features, and the temporal attention in the decoder measures the temporal ones. In each module, bi-directional LSTM (BiLSTM) is used to capture further nonlinear information.

The principal contributions of this paper are as follows:

1. A novel deep learning framework is proposed for short-term traffic speed prediction.
2. Temporal clustering is used to improve dataset partition for enhancing performance.
3. Two attention mechanisms are introduced to capture important spatio-temporal information.
4. The effectiveness of the proposed model is validated in two real-word traffic datasets.

## 2. Methodology

**Figure 1:**
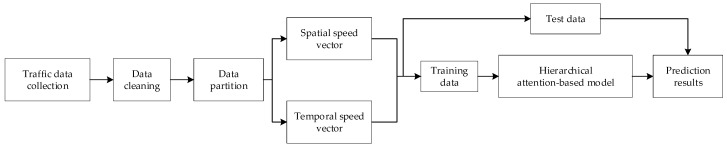
Framework of the proposed method.

With the overview of recent studies on short-term traffic speed prediction, a traffic speed prediction approach based on temporal clustering and hierarchical attention (TCHA) is proposed. Figure 1 shows the framework of this paper. We collected raw traffic speed data from cameras equipped on roads, capturing the passing vehicles and saving information into databases. Certain data cleaning methods were then employed to remove anomaly elements. The third step was to partition the pre-processed data into several clusters using a hierarchical temporal clustering algorithm. Traffic data in each cluster has a similar distribution, which can help improve the prediction accuracy. Following the above steps, two traffic speed vectors, which contain temporal speeds and spatial speeds respectively, were generated, and a hierarchical attention-based method was then applied to these two vectors to capture spatial and temporal features. In the encoder, spatial vectors were taken as inputs, and the relevance of each selected road was determined with the spatial attention. The hidden states computed from the encoder together with the temporal vectors were concatenated as inputs for the decoder. In the decoder, the importance of each time step was calculated with the temporal attention. Finally, a fully connected layer was used for prediction. Each part is detailed in the following subsections.

### 2.1. Data Partition

Traffic environments are changing every day, and some researchers [37] have proved that the traffic environment (or context) dimension is the most relevant to traffic prediction. The context includes the day of a week (weekday or weekend), emergency events (or how far it happens away from the target road), weather (rainy, sunny, etc.), and so on. Accuracy may be low if we take all of the pre-processed speed data as training or testing samples, this fact is quite evident, e.g., a model is highly likely to be unable to detect a dog if it is trained by thousands of cats and tens of dogs. However, traffic environments are complex. There is no clear boundary or auto-adjusted model to partition. In this paper, we apply an unsupervised method, temporal clustering (TC), to partition raw traffic data. Temporal clustering analysis uses hierarchical clustering to obtain several clusters, in which all of the traffic speed data have similar traffic variation patterns. Algorithm 1 illustrates the details of this part.

All of the historical traffic speed data are divided by days before clustering, as the input dataset sequence is **D** = ($d_{1}$, $d_{2}$, …, $d_{p}$) with $d_{i}\in {\mathbb{R}}^{q}$, where *q* represents the number of data in one day, and *p* is the number of samples, i.e., the number of initial cluster. Threshold *θ* and *sim_max* is first initialized. *sim_max* is a constant that represents the maximum value of an integer. In each loop, the similarity within clusters is calculated, and two clusters whose similarity obtains the maximum are aggregated. The whole procedure will be stopped when there is only one cluster, or the maximum similarity is less than the threshold. In this paper, the Pearson correlation coefficient [38] is employed as a similarity function. The data of each cluster can be used to train a prediction model. Before the prediction, the similarity between the current day’s data and each cluster is calculated, the closest cluster and its model are selected to predict the short-term traffic speed.

**Algorithm 1:** Temporal clustering analysis.

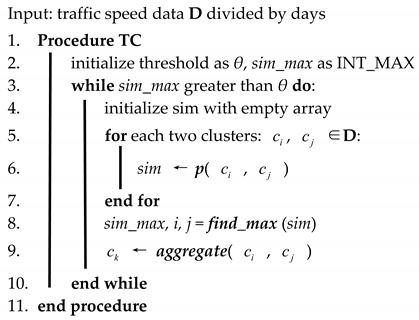



### 2.2. The Attention Model

The attention model is aimed to determine how strong the target road’s speed is relative to several time steps and the surrounding roads. As we know, the historical traffic speed data at a closer time step and the surrounding road will have a greater impact on future speed data [39,40], but the influence factor may differ in different cases.

**Figure 2:**
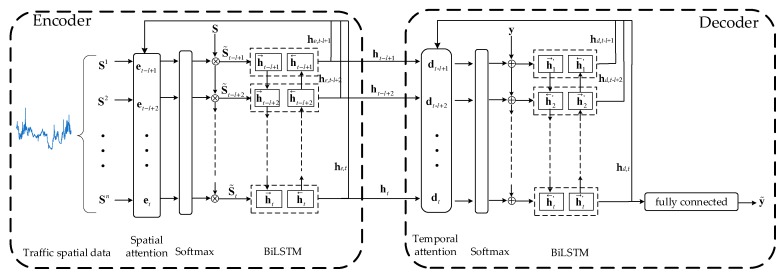
Graphic illustration of temporal clustering and hierarchical attention (TCHA).

There are two attention mechanisms in the model, i.e., the spatial attention and the temporal attention (see Figure 2). The spatial attention is used in the encoder to capture the spatial features and determine the importance of each space point with a BiLSTM network, and the temporal attention is applied in the decoder to capture temporal relations and decide the importance of each time step with another BiLSTM network.

#### 2.2.1. The Encoder Module

The encoder is essentially a BiLSTM network [41], aiming to determine the importance of each space point. Given the input spatial sequences at time step *t*, $S=(S_{t-l+1}^{},S_{t-l+2}^{},\ldots ,S_{t}^{}{)}^{T}$ $\in {\mathbb{R}}^{l\times n}$, where *n* is the number of selected surrounding roads, and *l* is the time lags. The spatial matrix can also be written as $S=(S_{}^{1},S_{}^{2},\ldots ,S_{}^{n})$, where $S_{}^{i}\in {\mathbb{R}}^{l}$ represents the speed matrix of road *i* at all time steps.

The spatial attention mechanism can be constructed by a soft attention mechanism:

(1) $$e_{t}^{i}=Z_{e}^{T}\tanh(W_{e}\left[h_{e,t-1};c_{t-1};S_{}^{i}\right]+b_{e})+b_{ze}^{i},$$

(2) $$\alpha_{t}^{i}=\frac{\exp(e_{t}^{i})}{\sum_{i=1}^{n}\exp(e_{t}^{i})},$$

where $\left[h_{e,t-1};c_{t-1};S_{}^{i}\right]\in {\mathbb{R}}^{2m+l}$ is a concatenation of the previous encoder hidden state, memory cell, and current spatial data, *m* is the encoder hidden size, $Z_{e}\in {\mathbb{R}}^{l}$ and $W_{e}\in {\mathbb{R}}^{l\times (2m+l)}$ are the weights of linear functions, $b_{e}\in {\mathbb{R}}^{l}$ and $b_{ze}\in {\mathbb{R}}^{n}$ are the bias terms, which are all the parameters to learn, $c_{t}$ and $h_{e,t}\in {\mathbb{R}}^{m}$ are memory cells and the linear transformation of hidden states in the encoder procedure, which are initialized as zero tensors and will be illustrated in detail, and tanh(·) is the hyperbolic tangent function. A SoftMax function is applied to compute the spatial attention weights $\alpha_{t}^{}\in {\mathbb{R}}^{n}$, which represent the scores of each selected road, with the higher score representing the stronger relation.

With the attention weights, the input spatial matrices can be transferred to

(3) $${\tilde{S}}_{t}=(\alpha_{t}^{1}S_{t}^{1},\alpha_{t}^{2}S_{t}^{2},\ldots ,\alpha_{t}^{n}S_{t}^{n}{)}^{T},$$

where ${\tilde{S}}_{t}$ contains spatial information. 

To extract further features and learn parameters, an activation function should be applied. In this paper, we use BiLSTM, which is specialized for sequence learning. BiLSTM contains the forward LSTM (denoted as $\vec{\mathrm{LSTM}}$), which processes spatial data from ${\tilde{S}}_{t-l+1}$ to ${\tilde{S}}_{t}$, and the backward $\overset{\leftarrow }{\mathrm{LSTM}}$ (LSTM), which processes spatial data from ${\tilde{S}}_{t}$ to ${\tilde{S}}_{t-l+1}$.

The outputs of BiLSTM can be expressed as follows:

(4) $${\vec{h}}_{t}=\vec{\mathrm{LSTM}}(w_{t1},{\vec{h}}_{t-1}),$$

(5) $${\overset{\leftarrow }{h}}_{t}=\overset{\leftarrow }{\mathrm{LSTM}}(w_{t2},{\overset{\leftarrow }{h}}_{t-1}),$$

(6) $$h_{t}=[{\vec{h}}_{t};{\overset{\leftarrow }{h}}_{t}],$$

(7) $$h_{e,t}=W_{e,t}h_{t}+b_{e,t},$$

where ${\vec{h}}_{t}\in {\mathbb{R}}^{m}$ represents the forward hidden state, and ${\overset{\leftarrow }{h}}_{t}\in {\mathbb{R}}^{m}$ represents the backward hidden state, both of which capture the deeper information of all inputs at time *t*. $h_{t}\in {\mathbb{R}}^{2m}$ is the concatenation of ${\vec{h}}_{t}$ and ${\overset{\leftarrow }{h}}_{t}$, which represents the encoder hidden state and will be decoded in temporal attention. $h_{e,t}\in {\mathbb{R}}^{m}$ is the linear transformation of $h_{t}$, which will be used for spatial attention calculation, $W_{e,t}\in {\mathbb{R}}^{m\times 2m}$ and $b_{e,t}\in {\mathbb{R}}^{m}$ are weight terms and bias terms.

With the proposed spatial attention, the encoder will focus more on several roads that obtain higher weights.

#### 2.2.2. The Decoder Module

Another BiLSTM network is used in the decoder to determine how strong each time step will influence the predicted traffic speed.

With the computed hidden states $\left(h_{t-l+1},h_{t-l+2},\ldots ,h_{t}\right)$ in the encoder layer, we calculate the temporal attention weights as follows:

(8) $$d_{t}^{i}=Z_{d}^{T}\tanh(W_{d}\left[h{}_{d,t-1}^{\prime };c{}_{t-1}^{\prime };h_{i}\right]+b_{wd})+b_{zd}^{i},$$

(9) $$\beta_{t}^{i}=\frac{\exp(d_{t}^{i})}{\sum_{i=1}^{l}\exp(d_{t}^{i})},$$

where $\left[h{}_{d,t-1}^{\prime };c{}_{t-1}^{\prime };h_{i}\right]\in {\mathbb{R}}^{2m+2k}$ is a concatenation of the previous decoder hidden state, memory cell, and encoder hidden state, *k* is the decoder hidden size, $W_{d}\in {\mathbb{R}}^{m\times (2m+2k)}$, $Z_{d}\in {\mathbb{R}}^{m}$, $b_{zd}\in {\mathbb{R}}_{}^{l}$, and $b_{wd}\in {\mathbb{R}}^{m}$ are the weights and the bias terms in the decoder, which are all the parameters to learn. The temporal attention weights represent how much each encoder hidden state will influence the prediction results. Since each encoder hidden state has contained the spatial factors, a context vector that represents the sum of all encoder hidden states can be computed in the attention mechanism:

(10) $$c_{t}^{}=\sum_{i=1}^{l}\beta_{t}^{i}h_{t-i+1}^{}.$$

Combined with context vectors, the input temporal sequence at time *t* $\left(y_{t-l+1}^{},y_{t-l+2}^{},\ldots ,y_{t}^{}\right)$ can be transferred to

(11) $${\tilde{y}}_{t}=W_{c}^{T}\left[\begin{array}{l} c_{t}\\ y_{t} \end{array}\right]+b_{c},$$

where $\left[\begin{array}{l} c_{t}\\ y_{t} \end{array}\right]\in {\mathbb{R}}^{2m+1}$ is the concatenation of the target road’s speed at time *t* and context vector; $W_{c}\in {\mathbb{R}}^{2m+1}$ and $b_{c}\in \mathbb{R}$ are parameters to learn, which help map the concatenation to new inputs in the decoder. Similarly, we apply BiLSTM to extract further features in the time dimension. The outputs can be expressed as follows:

(12) $${\vec{h}}_{t}^{\prime }=\vec{\mathrm{LSTM}}(w_{t1}^{\prime },{\vec{h}}_{t-1}^{\prime }),$$

(13) $${\overset{\leftarrow }{h}}_{t}^{\prime }=\overset{\leftarrow }{\mathrm{LSTM}}(w_{t2}^{\prime },{\overset{\leftarrow }{h}}_{t-1}^{\prime }),$$

(14) $$h_{t}^{\prime }=[{\vec{h}}_{t}^{\prime };{\overset{\leftarrow }{h}}_{t}^{\prime }],$$

(15) $$h_{d,t}=W_{d,t}h_{t}^{\prime }+b_{d,t},$$

where ${\vec{h}}_{t}^{\prime }\in {\mathbb{R}}^{k}$ represents the forward hidden state, and ${\overset{\leftarrow }{h}}_{t}^{\prime }\in {\mathbb{R}}^{k}$ represents the backward hidden state, both of which capture the deeper information of entire inputs at time *t*. $h_{t}^{\prime }\in {\mathbb{R}}^{2k}$ is the concatenation of ${\vec{h}}_{t}^{\prime }$ and ${\overset{\leftarrow }{h}}_{t}^{\prime }$, $h_{d,t}\in {\mathbb{R}}^{k}$ is the linear transformation of $h_{t}^{\prime }$, which will be used for temporal attention calculation, $W_{d,t}\in {\mathbb{R}}^{k\times 2k}$ and $b_{e,t}\in {\mathbb{R}}^{k}$ are weight terms and bias terms.

Finally, the prediction result can be iteratively computed through the fully connected layer:

(16) $$y_{pred}^{t+1}=W_{y}^{T}\left[\begin{array}{l} c_{t}\\ h_{t}^{\prime } \end{array}\right]+b_{y},$$

where $\left[\begin{array}{l} c_{t}\\ h_{t}^{\prime } \end{array}\right]\in {\mathbb{R}}^{2m+2k}$ is the concatenation of the context vector and the decoder hidden state, $W_{y}\in {\mathbb{R}}^{2m+2k}$, $b_{y}\in \mathbb{R}$ are parameters to learn, and $y_{pred}^{t+1}$ represents the predicted result at time step *t* + 1.

### 2.3. Model Optimization

As mentioned above, there are parameters to learn with training samples. The Adam [42] optimizer is used to train the model, and the mean square error in Equation (17) is employed as a loss function to measure the difference between the ground truth values and the predicted results:

(17) $$loss=\frac{1}{N}\sum_{i=1}^{N}{\left(y_{pred}^{i}-y_{true}^{i}\right)}^{2},$$

where $y_{pred}^{i}$ is the predicted result, $y_{true}^{i}$ is the ground truth value, and *N* denotes the number of training samples.

## 3. Results

In this section, the traffic speed data collected from Xiaoshan District, Hangzhou, China, are used to demonstrate the effectiveness of the proposed TCHA method, through comparing to several state-of-the-art prediction approaches with deep architectures.

### 3.1. Experimental Setup

**Figure 3:**
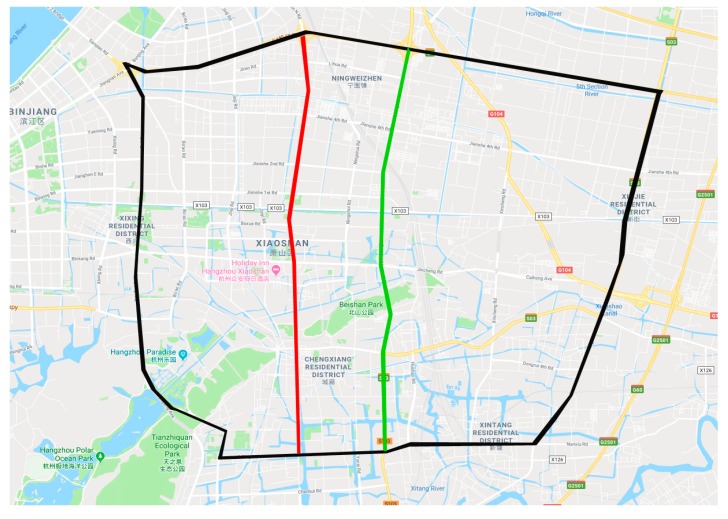
Traffic speed locations.

As is shown in Figure 3, to evaluate the performance of the proposed TCHA method, part of the Xiaoshan District was chosen to performance experiments (in black), Shixin Road (in red) and Tonghui Road (in green) were selected as target roads, and the remaining segments were used to determine the spatial features. There were 38 detectors along the whole roads, and the traffic speed data were collected and calculated every 5 min. Consequently, one detector preserved 288 data per day. As mentioned before, we partitioned the raw traffic speed data into two vectors; however, due to the malfunction of detectors and the failure of data transmission, there were some incorrect data, which includes the following:

- Missing data. There are some zero elements in the raw data, which are marked as missing data.
- Outliers. Considering that the speed limits in Hangzhou are usually lower than 80 km/h, we set the maximum traffic speed as 100 km/h, which means that, if a certain record of speed is higher than the threshold, it is marked as an outlier.
- Noisy data. Since it is a real-world traffic speed dataset, dramatic changes should be avoided. Consequently, traffic speeds differing more than 20 km/h between two adjacent time points are considered as noisy data.

The traffic speed data marked as any type of anomaly data are replaced by the average speed of the previous 10 min. In general, the proportion of anomaly records is less than 10%.

**Figure 4:**
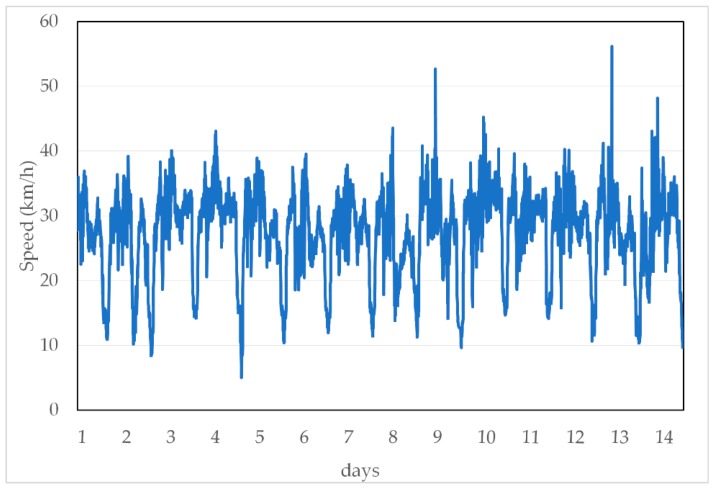
Traffic speed patterns.

**Figure 5:**
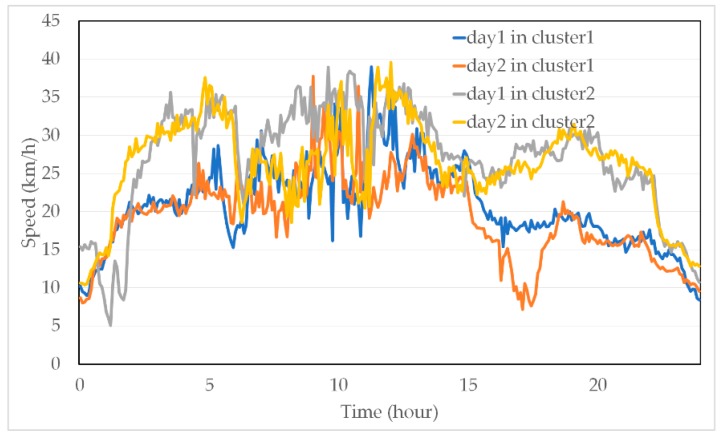
Distribution of traffic speed data in different clusters.

Figure 4 is a plot of several typical traffic speeds over time, which demonstrates obvious period patterns. Different distributions of traffic speed data in different clusters after employing TC can be seen in Figure 5. It is clear that the traffic speed data in the same cluster have similar distributions, while the distribution may differ more when the data come from two clusters.

The TCHA method is evaluated on different prediction horizons, and the time lag *l* is set as 12. Prediction horizons are set up to 5, which means that 60 min of historical traffic speed data are used to predict speed of the following 25 min. For example, suppose the current time is 7:00 a.m.: The proposed method will predict the speed of 7:05 a.m., 7:10 a.m., 7:15 a.m., 7:20 a.m., and 7:25 a.m.

The proposed TCHA method is compared to several state-of-the-art prediction approaches, which includes the following:

- support vector regression (SVR) [43], which uses linear support vector machine for regression tasks, especially time-series prediction;
- stacked autoencoder (SAE) [21], which encodes the inputs into dense or sparse representations by using multi-layer autoencoders;
- long short-term memory (LSTM) [44], which is an extension of recurrent neural networks (RNNs) and has an input gate, a forget gate, and an output gate so as to deal with the long-term dependency and gradient vanishing/explosion problems;
- the gated recurrent unit (GRU) [44], which has an architecture similar to LSTM but only has two gates, a reset gate, and an update gate, which makes GRU have fewer tensor operations than LSTM;
- the hierarchical attention model (HA), which uses spatial and temporal attention mechanisms to capture spatial and temporal features respectively, but TC is not employed to the input data, and it is different from the proposed TCHA.

To guarantee the fairness of experiments, all of the approaches are trained by the Adam optimizer, which updates the parameters with a gradient descent algorithm, and the batch size is set as 128. The threshold *θ* of TC is another hyper-parameter, which is set as 0.6 [36]. All of the neural networks are built in the pyTorch framework. For support vector regression, radial basis function (RBF) is applied as a kernel function for its better performance in a non-linear situation.

### 3.2. Evaluation Criteria

The mean absolute error (MAE), mean relative error (MRE), and the root-mean-squared error (MRSE) are employed as the evaluation criteria to evaluate the prediction accuracy, which are defined as follows:

(18) $$\mathrm{MAE}=\frac{1}{n}\sum_{i=1}^{n}\left|y_{pred}^{i}-y_{true}^{i}\right|,$$

(19) $$\mathrm{RMSE}=\sqrt{\frac{1}{n}\sum_{i=1}^{n}{\left(\left|y_{pred}^{i}-y_{true}^{i}\right|\right)}^{2}},$$

(20) $$\mathrm{MRE}=\frac{1}{n}\sum_{i=1}^{n}\left|\frac{y_{pred}^{i}-y_{true}^{i}}{y_{true}^{i}}\right|,$$

where *n* denotes the number of prediction samples, $y_{pred}^{i}$ is the prediction value, and $y_{true}^{i}$ is the true value. MSE and MAE can measure the absolute differences between the predicted and true values, and MRE can measure the relative ones.

### 3.3. Experiments Result and Analysis

Table 1 and Table 2 show the errors of different prediction horizons using different methods for both target roads. The outperformance of the proposed TCHA method can be seen at all time points.

On Shixin Road, compared to the hierarchical attention model without TC, the TCHA has average improvements of 0.1029, 0.2369, and 0.0063 on MAE, RMSE, and MRE, and outperforms the relative accurate method. The GRU showed improvements of 0.5360, 0.496 and 0.0919 on MAE, RMSE, and MRE. On Tonghui Road, the proposed TCHA model outperforms the HA with improvements of 0.2353, 0.2370, and 0.0082 on MAE, RMSE and MRE.

With the increase in prediction horizons, the errors become larger, whereas the TCHA still reaches the best performance, which implies that long-term prediction is more difficult and challenging, and confirms that extracting temporal and spatial features in encoder and decoder mechanisms is reasonable.

**Figure 6:**
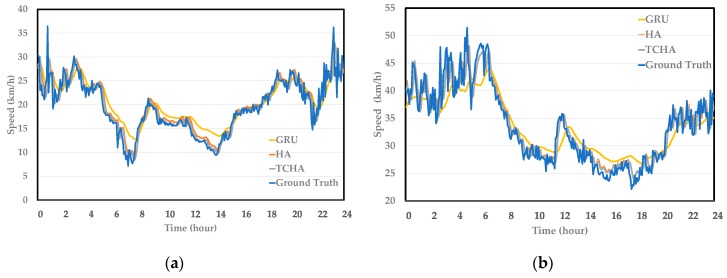
Traffic speed prediction performance. (**a**) Shixin Road and (**b**) Tonghui Road.

Figure 6 shows the curves of the predicted results corresponding to the ground truth on both roads. Different colors represent different algorithms, during the entire day. The proposed TCHA algorithm fits the true speed data whether it is peak hour or not. In Figure 6b, the difference between the ground truth and the results of the proposed method is relatively large around 15 h, which may be due to the large variation when traffic speed meets peak hour. Peak hour and emergencies are challenges when making short-term predictions. The large influence can be seen more clearly when using GRU to make prediction. GRU also fits the general trend of traffic speed in one day, but when traffic speed meets large variation, the prediction performance gets lower, which further emphasizes the importance of spatial and temporal mechanisms, and demonstrates that our proposed model is good approximate to the ground truth.

**Figure 7:**
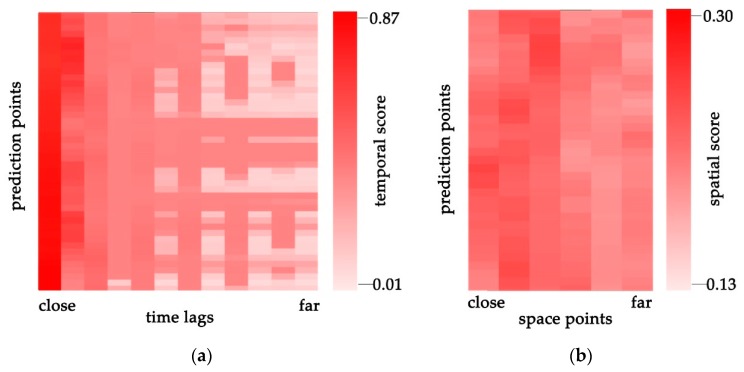
Attention visualization of average score on different prediction points at (**a**) different time lags and (**b**) different space points.

Figure 7 shows the attention scores learned by the hierarchical attention mechanism described in Section 2.2. The darker color indicates a higher importance when making predictions, while the lighter part indicates a lower importance. Rows represent the parts of all the prediction points, and the columns in the two subfigures represent the time lag and space point importance, respectively. In the temporal dimension, closer time lags contribute more to prediction, which matches our intuition that average traffic speed is always continuous and will not have dramatic changes compared to the speed at adjacent times. When time lag is 1, the temporal score is always highest, which may reach 0.87 in some cases. Generally, traffic speed at the farthest time lag (more than 50 min) has little or almost no contribution to future prediction. While in the spatial dimension, it is harder to find the obvious and significant regularity, the difference between the largest attention score and the lowest score is only 0.17. However, the proposed TCHA model still achieves higher weights with respect to the upstream and downstream roads than with respect to the indirect surrounding roads.

## 4. Conclusions

In this paper, we propose a traffic speed prediction approach based on temporal clustering and hierarchical attention (TCHA) for traffic speed prediction. We first divided the historical traffic speed data into several clusters based on the similarity function of the Pearson correlation coefficient. A spatial attention mechanism was then designed in the encoder, which can adaptively select the relatively important segments for prediction, and a temporal attention mechanism was used in the decoder to determine the importance of each time step. The BiLSTM network was used in both mechanisms, for its better ability to capture further features in sequence learning. To validate the effectiveness of the proposed model, five approaches (i.e., SVR, SAE, LSTM, GRU, and the original hierarchical attention model) are compared to the TCHA based on the same dataset. The experiment results confirm that our proposed method can learn more temporal and spatial information to increase prediction accuracy. Several findings can be summarized:

1. With the increasement of prediction horizons, the prediction errors become larger.
2. Making traffic speed prediction during peak hour is challenging.
3. Although GRU can be regarded as a simplification of LSTM, it can reach a higher accuracy in most cases.

In the future, the model can be improved by considering some environmental factors, social events, and especially traffic signal control data. Due to the faults of devices and transmission, missing data and anomaly data will decrease the accuracy of the trained model and predicted results. It is necessary to generate data from various sources, i.e., camera detectors, GPSs, taxies, etc., and to apply data fusion methods to improve the confidence of the raw traffic speed data.

## Figures and Tables

**Table 1:** Performance of difference prediction horizons (Shixin Road).

Algorithm	Error Index	Horizon 1	Horizon 2	Horizon 3	Horizon 4	Horizon 5
SVR	MAE	3.1410	3.2829	3.4212	3.5746	3.7013
	RMSE	4.4425	4.5538	4.6558	4.7810	4.8937
	MRE	0.1670	0.1750	0.1830	0.1925	0.2004
SAE	MAE	2.2477	2.8831	3.0239	3.1550	3.2762
	RMSE	3.0273	3.6742	3.8646	4.0222	4.1691
	MRE	0.1226	0.1627	0.1695	0.1761	0.1823
LSTM	MAE	2.4501	2.7280	2.8957	3.0512	3.1998
	RMSE	3.2971	3.5613	3.7749	3.9642	4.1405
	MRE	0.1283	0.1464	0.1553	0.1635	0.1715
GRU	MAE	2.1859	2.5738	2.7458	2.9019	3.0501
	RMSE	2.9850	3.4393	3.6518	3.8358	4.0064
	MRE	0.1166	0.1378	0.1464	0.1544	0.1621
HA	MAE	1.6259	2.0841	2.3489	2.5223	2.7112
	RMSE	2.3756	3.0004	3.2993	3.5420	3.7166
	MRE	0.0768	0.1029	0.1163	0.1259	0.1374
TCHA	MAE	1.5051	2.0017	2.1689	2.3892	2.7127
	RMSE	2.3040	2.8217	3.1351	3.4294	3.7481
	MRE	0.0681	0.0984	0.1063	0.1182	0.1366

**Table 2:** Performance of difference prediction horizons (Tonghui Road).

Algorithm	Error Index	Horizon 1	Horizon 2	Horizon 3	Horizon 4	Horizon 5
SVR	MAE	4.9455	5.0361	5.1511	5.2186	5.2512
	RMSE	6.4193	6.4726	6.5692	6.6283	6.6590
	MRE	0.1496	0.1525	0.1558	0.1577	0.1586
SAE	MAE	3.5900	3.6890	3.8020	4.7404	4.9129
	RMSE	4.2695	4.4256	4.5911	6.0130	6.2097
	MRE	0.1163	0.1191	0.1223	0.1428	0.1482
LSTM	MAE	3.1010	3.2402	3.6749	3.7564	4.0615
	RMSE	4.0861	4.2574	4.6404	4.7457	5.0561
	MRE	0.0935	0.0977	0.1143	0.1167	0.1280
GRU	MAE	2.8053	2.9635	3.2987	3.5628	3.6247
	RMSE	3.8097	4.0104	4.2109	4.4912	4.6136
	MRE	0.0826	0.0872	0.1022	0.1112	0.1221
HA	MAE	1.8842	2.7023	2.9582	3.1797	3.3378
	RMSE	2.5605	3.6264	3.9319	4.1614	4.3393
	MRE	0.0554	0.0806	0.0887	0.0960	0.1012
TCHA	MAE	1.7686	2.4112	2.7111	2.9115	3.0833
	RMSE	2.4271	3.3031	3.6741	3.9253	4.1050
	MRE	0.0518	0.0706	0.0802	0.0865	0.0916
